# A Brief Narrative Review of the Underlying Mechanisms Whereby Omega-3 Fatty Acids May Influence Skeletal Muscle: From Cell Culture to Human Interventions

**DOI:** 10.3390/nu15132926

**Published:** 2023-06-28

**Authors:** Maryam Taheri, Philip D. Chilibeck, Stephen M. Cornish

**Affiliations:** 1 Faculty of Sport Sciences and Health, Shahid Beheshti University, Tehran 19839 69411, Iran; maryam.taheri1372@yahoo.com; 2 Faculty of Kinesiology and Recreation Management, University of Manitoba, Winnipeg, MB R3T 2N2, Canada; 3 College of Kinesiology, University of Saskatchewan, Saskatoon, SK S7N 5A9, Canada; phil.chilibeck@usask.ca; 4 Centre on Aging, University of Manitoba, Winnipeg, MB R3E 0J9, Canada

**Keywords:** omega-3, sarcopenia, anabolic, mechanism

## Abstract

Skeletal muscle is essential for human locomotion as well as maintaining metabolic homeostasis. Age-related reduction in skeletal muscle mass, strength, and function (i.e., sarcopenia) is a result of pathophysiological processes that include inflammation, alteration of molecular signaling for muscle protein synthesis and degradation, changes in insulin sensitivity, as well as altered skeletal muscle satellite cell activity. Finding strategies to mitigate skeletal muscle loss with age is deemed paramount as the percentage of the population continues to shift towards having more older adults with sarcopenia. Recent research indicates omega-3 fatty acid supplementation can influence anabolic or catabolic pathways in skeletal muscle. Our brief review will provide a synopsis of some underlying mechanisms that may be attributed to omega-3 fatty acid supplementation’s effects on skeletal muscle. We will approach this review by focusing on cell culture, animal (pre-clinical models), and human studies evaluating omega-3 fatty acid supplementation, with suggestions for future research. In older adults, omega-3 fatty acids may possess some potential to modify pathophysiological pathways associated with sarcopenia; however, it is highly likely that omega-3 fatty acids need to be combined with other anabolic interventions to effectively ameliorate sarcopenia.

## 1. Introduction

There is a great deal of interest surrounding the maintenance and improvement of skeletal muscle mass and strength using a wide variety of interventions. In younger adults, improving skeletal muscle mass and strength may be important for the performance-enhancing benefits it can bestow in athletic or recreational activities. In older adults, finding strategies to reduce the consequences of sarcopenia (i.e., low muscle mass, low muscle strength, low functional ability) is deemed paramount to reducing the morbidity and mortality associated with this condition [1]. Delineation of the different interventions that can be used in the aging population to reduce skeletal muscle and strength losses is believed to be one of the most pressing healthcare issues currently facing society [2]. While there are many different types of interventions (exercise, pharmaceutical, nutritional) that could be used to help delay muscle strength and mass loss, one nutritional intervention that has received attention recently is the use of omega-3 (ω-3) fatty acid supplementation [3] to help stimulate anabolic activity or reduce catabolic activity in skeletal muscle.

Omega-3 fatty acids are polyunsaturated fatty acids with a double-bond three atoms away from their methyl end. The most predominant forms of ω-3 fatty acids that have been investigated for their effects on muscle include docosahexaenoic acid (DHA), eicosapentaenoic acid (EPA), and alpha-linolenic acid (ALA). These ω-3 fatty acids incorporate into cellular phospholipid membranes and produce physiological effects via their ability to produce various eicosanoids (i.e., leukotrienes, prostaglandins, prostacyclins, and thromboxanes) via the cyclooxygenase and lipoxygenase enzymatic pathways. Typically, the traditional Western diet is composed of excessive ω-6 fatty acids (e.g., linoleic acid), which may produce a more inflammatory environment if not balanced properly with a greater intake of ω-3 fatty acids [4]. This may be one reason why older adults may be affected by chronic low-grade inflammation (i.e., ‘inflammaging’). The anti-inflammatory function of ω-3 fatty acids is needed to reduce the risk of developing chronic diseases and to balance the ratio of ω-6:ω-3 fatty acids [5]. Inflammaging may result in the deterioration of skeletal muscle mass through the release of cytokines (e.g., tumor necrosis factor-α (TNF-α)), stimulating the process of myocyte destruction via apoptotic pathways [6]. Supplementing older adults with ω-3 fatty acids (or ingesting more ω-3s within the diet) to change the ω-6:ω-3 ratio may be effective for reducing low-grade inflammation [7].

**Figure 1:**
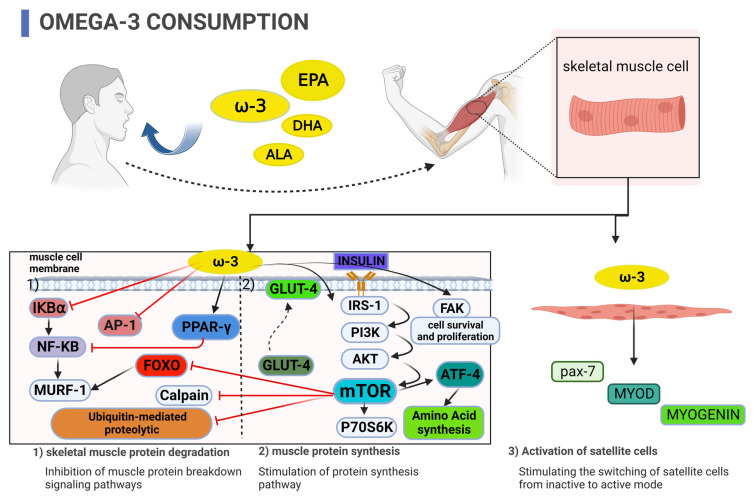
Potential mechanisms by which supplementation with omega-3 fatty acids inhibit skeletal muscle protein degradation, stimulate muscle protein synthesis, enhance insulin sensitivity, and activate skeletal muscle satellite cells.

The mechanisms likely involved in the anabolic or anti-catabolic effects of ω-3 fatty acid supplementation revolve around (1) inflammatory milieu modification, (2) activation of the mechanistic target of rapamycin (mTOR) pathway in skeletal muscle, (3) improved insulin sensitivity, and (4) the potential to alter skeletal muscle satellite cell activity (see Figure 1) [8,9,10,11,12]. This narrative review describes the mechanisms whereby ω-3 fatty acids increase skeletal muscle hypertrophy or prevent muscle catabolism. We will focus our review on cell culture, animal, and human studies that have used ω-3 supplementation strategies alone to affect skeletal muscle.

## 2. Cell Culture Studies

Studies with cell cultures have determined how ω-3 fatty acids may affect pathways associated with inflammation, insulin sensitivity, and synthesis of muscle protein. EPA and DHA have been the most predominantly utilized fatty acids for the incubation of C2C12 cells (i.e., mouse skeletal myoblast cells) for in vitro studies. EPA and DHA are the fatty acids that are thought to produce the greatest physiological effects. 

One way in which skeletal muscle-induced protein degradation may occur is through chronically elevated inflammatory milieu via TNF-α stimulated apoptosis of myocytes [13]. The nuclear factor kappa-light-chain-enhancer of activated B-cells (NF-κB) is a transcription factor that, when activated, induces a strong pro-inflammatory response. The nuclear factor of kappa light polypeptide gene enhancer in B-cells inhibitor, alpha (IκBα), is an important inhibitor of NF-κB. In C2C12 myoblasts, DHA treatment was more effective for inhibiting protein degradation when compared to EPA, and DHA was able to decrease the phosphorylation of IκBα (i.e., the inhibitor of NF-κB) and increase the protein content of IκBα more so than EPA [14]. Further, in C2C12 myotubes, EPA, but not ALA, was able to decrease IκBα phosphorylation and increase total protein IκBα levels which reduced NF-κB DNA-binding activity [15]. This study also demonstrated that EPA incubation was able to reduce mRNA for muscle RING finger 1 (MuRF1), an enzyme that stimulates the degradation of myosin heavy chains in muscle when tested against a control (bovine serum albumin), as well as enhance mRNA for the transcription factor peroxisome proliferator-activated receptor-γ (PPAR-γ) [15]. The authors concluded that EPA was able to effectively inhibit the IκBα/NF-κB/MuRF1 pathway via PPAR-γ. Additional results in C2C12 myoblasts incubated with TNF-α demonstrated that introducing EPA to the media decreased the effects of TNF-α on apoptosis and improved myotube formation by suppressing the inflammatory environment created by the activation of NF-κB pathways via TNF-α stimulation [16,17]. This suggests that one mechanism by which ω-3 fatty acids may enhance skeletal muscle is through a dampening of NF-κB pathway activation, which could promote inhibition of skeletal muscle protein degradation. Thus, it seems that EPA and DHA can effectively dampen the NF-κB pathway; however, ALA may be less effective at modifying this pathway, at least in cell culture. Interestingly, doses of EPA/DHA/ALA with ranges from 150–700 µM were used to incubate cells, and it seems that moderate-high concentrations of ω-3 DHA and EPA (i.e., 300–700 µM) decreased protein degradation more efficiently [14,15]. However, physiological concentrations in young, healthy adults, without supplementation, range from 12.0–186.9 µmol/L for ALA and 7.2–237.5 µmol/L for DHA, thus, not necessarily reaching the levels used in cell culture to stimulate pathways that may enhance skeletal muscle anabolism [18]. Nevertheless, the physiological concentrations of a combined 3 g of EPA and DHA supplement that is bound to ethyl ester or monoacylglycerol can result in a peak concentration of between 318–858 µmol/L, respectively, which may be enough to enhance skeletal muscle effects [19]. 

Myogenic regulatory factors (MRFs) are a group of transcription factors that control myogenesis during activation, proliferation, and differentiation of satellite cells [20] in postnatal tissue and include Myf5, MyoD, myogenin, and MRF4 [21]. Satellite cells are important muscle stem cells that can be incorporated into muscle fibers to form new myonuclei to stimulate protein synthesis [20]. In mouse myoblasts, treatment of cells with a common saturated fatty acid (palmitate) causes cell death, but EPA was able to effectively preserve cell viability via inhibition of mitogen-activated protein kinase (MAPK) apoptosis as well as stimulate MyoD, thus potentially reducing catabolic activity and increasing anabolic activity in skeletal muscle [22]. In another study, DHA was able to produce myotube hypertrophy when mouse myoblasts were incubated with palmitate and DHA together, while palmitate incubation alone caused significant atrophy of the cells [23]. Also, in C2C12 cells, the incubation and incorporation of DHA into the phospholipid membrane was able to enhance the resilience of the cell membrane to mechanical stress and, thus, may reduce cell breakage during a mechanical contraction when compared to arachidonic acid (a predominant ω-6 fatty acid) [24]. 

C2C12 myoblasts were incubated with lipopolysaccharide (LPS, which are components of membranes of toxic bacteria), inducing deficiency in insulin signaling, but when the myoblasts were co-incubated with EPA, there was a restoration of insulin signaling [25]. This study further demonstrated the potential effect that EPA might have in preserving phosphorylation of mammalian target of rapamycin (mTOR), a pathway important for activation of translation and, therefore, muscle protein synthesis when cells are facing an LPS challenge [25]. In this study, NF-κB and activation protein-1 (AP1, a transcription factor controlling programed cell death, i.e., apoptosis) were inhibited by EPA, thus providing a potential mechanism whereby mTOR phosphorylation could persist even when facing an LPS challenge [25]. Further study in C2C12 myoblasts indicated that DHA was able to rescue palmitate-induced atrophy by promoting protein kinase (Akt) activation, which inhibits the transcription factor forkhead box O-3 (FoxO3), important for apoptosis [26]. Interestingly, a different study in C2C12 myotubes found that EPA incubation promoted protein synthesis and lessened protein breakdown; however, DHA had no effect on the synthesis or breakdown of proteins in the cell culture [27]. The two aforementioned studies differed in terms of study design (i.e., one included palmitate incubation while the other incubated only with EPA or DHA), and thus, this may explain the discrepancy in the results.

These cell culture studies have been performed under conditions that likely optimize ω-3 fatty acids’ effectiveness. Even though cell culture studies are important to help understand underlying mechanisms, the in vivo effects may be less pronounced given the physiological milieu of intact organisms. In the next section, we review studies of animals evaluating the effectiveness of supplementation with ω-3 fatty acids.

## 3. Animal Studies

While pre-clinical animal-based study models are important for testing hypotheses and determining outcomes, currently, there is limited animal data (especially in healthy animals) on supplementing with ω-3 fatty acids for affecting skeletal muscle. The following section delineates the effects of supplementing with ω-3 fatty acids on skeletal muscle in mostly obese mouse and rat models, models that most likely potentiate muscle atrophy. Obesity is known to produce many metabolic disturbances, which may be attenuated by ω-3 intake, but does not necessarily represent a healthy condition to test the effectiveness of ω-3 fatty acid supplementation on skeletal muscle [28].

In an obese male mouse model, chia oil (which is high in ALA) was fed to animals who were also fed a diet high in fat to evaluate the effects on insulin signaling and fat and lean tissue mass [29]. Fat mass was reduced, and lean mass improved in the mice that consumed the chia oil when compared to the ones that only consumed the high-fat diet [29]. Further, there was an improvement in insulin signaling, glucose tolerance, insulin sensitivity, as well as glucose transport protein-4 translocation to the cell membrane (i.e., GLUT-4 translocation, important for glucose transport into the muscle) in the mice who received the chia oil [29]. The enhancement of insulin sensitivity and insulin signaling via the increased amount of ω-3 ALA in the diet would likely increase the signaling for cell growth (anabolism) and cell survival and proliferation through insulin-mediated activation of the mTOR pathway [30]. Another study performed in obese, type-2 diabetic Swiss mice demonstrated that flaxseed oil (high in ALA) combined with a high-fat diet was able to effectively reduce cytokines involved in inflammation and increase insulin receptor substrate-1/Akt phosphorylation when compared to the high-fat diet alone [31], thus confirming the previous study’s results. However, in an obese Zucker rat model, flaxseed oil (high in ALA) at 10% of the diet negatively altered insulin sensitivity, whole-body glucose homeostasis, and increased reactive oxygen species formation in the electron transport chain, which can lead to cell membrane damage and inflammation [32]. The interesting part of this study was the fact that the animals were exercising (treadmill running) while consuming the high ALA diet. Thus, there was a negation of the effectiveness of exercise for positively affecting insulin and glucose homeostasis with the high ALA diet. Additionally, the increase in ROS is troubling, given that this is linked to reduced skeletal muscle mass [33] and thus could perpetuate sarcopenia. Nevertheless, this animal model used a high amount of ALA in the diet, which is not likely typical in most human diets. The authors of the study provide a caveat in their manuscript, which states that achieving a dietary intake of ALA that represents 10% of the human diet is virtually impossible [32]. In fact, the authors point out that even supplementing the diet with 3 g/day of ω-3 fatty acids only represents 0.02% of daily fat intake if adopting a daily dietary intake for the fat of 40% [32].

In a rat model (13 months old), 8 weeks of supplementation with an EPA and DHA blend of ω-3 fatty acids was able to increase the phosphorylation of phosphoinositide 3-kinase (PI3K) and ribosomal protein S6 kinase (p70s6k), two important upstream and downstream components, respectively, of the Akt/mTOR pathway in the longissimus dorsi but not the soleus muscle [34]. Accordingly, it appears that the EPA/DHA blend used in this study may have muscle fiber type-specific effects as the longissimus dorsi is predominantly type-II muscle fibers while the soleus muscle is type-I. This may be translatable to a human model of sarcopenia as it is known that type-II muscle fibers predominantly are decreased with aging [35]; however, this would need to be tested directly in humans to confirm the results from the animal study. Further, a study in nephrectomized rats indicated that the addition of ω-3 fatty acids in the diet was able to regulate peroxisome proliferator-activated receptor gamma coactivator-1 alpha (PGC-1α) and, in turn, attenuate catabolism in skeletal muscle in this experimental chronic kidney disease model [36].

In summary, there are potential advantages and disadvantages to skeletal muscle in using various types of ω-3 fatty acids in animal models. In the two obese mouse models explained above, high amounts of ALA were able to rescue cell signaling that may enhance outcomes for skeletal muscle anabolism [29,31]. However, in the obese rat model, ALA showed negative consequences in terms of skeletal muscle, while the healthy rat model was able to show EPA/DHA supplementation effects were able to enhance certain aspects of molecular pathways of skeletal muscle anabolism, at least in type-II muscle fibers [32,34]. More research on the underlying effects of various ω-3 fatty acids on skeletal muscle is necessary to promote a greater understanding of the mechanisms whereby they may be effective for reducing skeletal muscle loss.

## 4. Human Studies

Several clinical trials in humans have evaluated EPA and DHA supplementation on skeletal muscle mass, strength, and functional ability [37,38,39,40,41,42,43,44]. The results of these studies indicate discrepancies between various study designs where supplementation with ω-3 fatty acids either positively influenced skeletal muscle parameters or had no effect. However, the majority of the studies that utilized an EPA/DHA supplementation mixed dosing strategy alone indicate increases in skeletal muscle function and size [37,41,42,44], no change in muscle size or lean tissue mass after 2 weeks of immobilization [39], or improvement in walking speed [43] after the combined EPA/DHA supplementation period. Nonetheless, there are also studies that failed to show the effectiveness of combined EPA and DHA supplementation on skeletal muscle strength or mass [38,40,45] as well as functional ability in older adults [40,45], and our ALA supplementation study in older adults was mostly ineffective at altering these parameters as well [46]. A recent study has indicated that IL-6 and subjective muscle soreness were attenuated with omega-3 supplementation at 3 g/day for 4 weeks before the muscle-damaging exercise protocol (downhill running) [47]. This reveals the potential benefit of using ω-3 fatty acids in the prevention of some components of exercise-induced muscle damage. Further, a randomized controlled trial that evaluated an ω-3 fatty acid-enriched diet with high whey protein supplementation, as well as vibration/home-based resistance exercise, found that IGF-1 was increased, inflammation decreased, and muscle power increased more so in the group with ω-3 supplementation; however, based on sex-analyses, the male group was the only group that showed effects on inflammation and muscle power [48]. It is likely that the differences noted in the above studies are due to alterations in the length of the supplementation period, the dose of EPA/DHA supplementation, the various parameters measured, and the type of study design used.

Oxylipins are a group of key mediators of the metabolism of long-chain polyunsaturated fatty acids in humans. A recent study determined the relationship between ω-3, ω-6, and ω-9 oxylipin metabolites and markers of skeletal muscle biology (skeletal muscle mass, strength, and functional performance) both before and after 24 weeks of supplementation with combined EPA, DHA, and docosapentaenoic acid (DPA) ω-3 fatty acids [49]. There was a negative correlation between ω-6 and ω-9 fatty acid metabolites with skeletal muscle mass and strength at baseline; however, even when the ω-3 fatty acid metabolites were increased after the 24-week supplementation period, there was no correlation with the ω-3 fatty acid oxylipins and skeletal muscle parameters [49]. The authors concluded that oxylipin status might have little to do with the health of skeletal muscle in humans of old age and low muscle mass.

Some human-based studies have attempted to identify the mechanism(s) underlying the positive effect of supplementing with ω-3 fatty acids on skeletal muscle mass and function. Integrated myofibrillar protein synthesis (MyoPS) remained higher in a group of healthy young females who supplemented with a combined EPA/DHA dose of 5 g/day for 4 weeks before undergoing 2 weeks of limb immobilization and then 2 weeks of recovery from the immobilization when compared to a placebo [50]. This resulted in an attenuation of muscle disuse atrophy in the ω-3 supplemented group (8% decrease) when compared to the control group (14% decrease) [50]. Further, activating transcription factor-4 (a factor involved in amino acid synthesis) was elevated in the ω-3 supplemented group, thus suggesting a potential mechanism whereby supplementing with ω-3 fatty acids may aid in the maintenance of skeletal muscle during disuse atrophy [50]. Another study in healthy older adults evaluated 24 weeks of combined EPA/DHA supplementation (2.16 g/day) on thigh muscle volume and various skeletal muscle genes involved in muscle structure and growth [51]. Here, they demonstrated significant increases in skeletal muscle mass and function as well as an attenuation of the inhibition of the mTOR pathway with supplementation of ω-3 fatty acid via inhibition of the calpain- and ubiquitin-mediated proteolytic pathways [51]. These results suggest that supplementation with ω-3 fatty acids in older humans may have a small but noticeable effect on the mTOR signaling pathway of skeletal muscle protein synthesis. This finding contrasts with the earlier findings of McGlory et al. [50], where there was no change in the phosphorylation of mTOR or the downstream product p70s6k in immobilized limbs of younger females after ω-3 supplementation. This may be due to the different ages of the participants and/or the differences in the two study designs (immobilization versus normal day-to-day activities). Nonetheless, in younger healthy, recreationally active young males, 4 weeks of supplementation with a combined EPA/DHA (4.4 g/day) blend was able to significantly increase focal adhesion kinase (which regulates cell survival and proliferation) from baseline to the 4-week time point in skeletal muscle biopsies [52]. In this study, the protein content of mTOR increased after the first week of ω-3 supplementation, suggesting an effect on this pathway in skeletal muscle [52]. Interestingly, in a series of two studies, Smith et al. [53,54] found that when combined with insulin and amino acid infusion, supplementation with the ω-3 fatty acids EPA/DHA (3.36 g/day) for 8 weeks improved synthesis rates for muscle protein as well as various components of the mTOR pathway in both young and old adults. These results indicate there may need to be other anabolic stimuli present to enhance the ability of ω-3 fatty acids to produce an anabolic effect in skeletal muscle. 

More recent studies have evaluated the effects of ω-3 supplementation strategies on skeletal muscle in younger individuals as well. Relative and absolute upper body strength and relative lower body strength were enhanced following 10 weeks of resistance exercise when it was combined with an EPA and DHA supplementation protocol when compared to a placebo supplement; conversely, body composition was improved with the resistance exercise but was not different between the ω-3 and placebo groups [55]. Another study suggests an attenuation of decreases in range of motion and creatine kinase blood concentrations with 4 weeks of supplementation (combined EPA and DHA) after participants were subjected to a 60-repetition eccentric muscle action exercise using the biceps brachii muscles [56]. Further research has indicated that 6 weeks of resistance-exercise training combined with EPA/DHA supplement was able to effectively reduce the inflammatory, muscle damage, and redox state in response to strenuous acute resistance exercise versus placebo in a young, healthy cohort [57]. Thus, in younger adult cohorts, it seems ω-3 may have a positive effect on some parameters associated with or directly linked to skeletal muscle function.

Some population-based research has been carried out evaluating ω-3 intake on strength and all-cause mortality. First, a cross-sectional study was conducted assessing ω-3 fatty acid intake in a large cohort of Korean adults, demonstrating that increased ω-3 intake was positively associated with hand grip strength suggesting an effect on skeletal muscle strength [58]. Other research indicated that data from the China Health and Nutrition Survey (CHNS) and the National Health and Nutrition Examination Survey (NHANES) showed varying results when evaluating dietary intake of ω-6 and ω-3 fatty acids [59]. Here, the researchers determined that the CHNS data indicates that increased dietary intake of marine ω-3 was inversely associated with mortality; however, the NHANES data specified that this relationship was not present [59]. Furthermore, the intake of ALA was positively associated with all-cause mortality in the CHNS data, whereas in the NHANES cohort, the relationship between ALA intake and all-cause mortality was inverse, suggesting differing results dependent on the population studied [59]. 

In summary, some research in humans that have utilized ω-3 supplementation strategies alone indicates a potential role for these fatty acids in maintaining or improving skeletal muscle outcomes in both younger and older adults. As the studies by Smith et al. [53,54] point out, the presence of another type of anabolic stimulus may be necessary to promote the positive effects of ω-3 intake. Certainly, there are other human-based studies that have evaluated supplementation of ω-3 fatty acids during resistance-exercise training programs [45,46,60,61,62,63,64,65,66,67] with varying effects on skeletal muscle mass, strength, and function. A recent meta-analysis has indicated that a longer (6-month) intervention is likely needed at a dose higher than 2 g/day to contribute to small muscle mass gain, strength increases, and functional improvements in older adults [68]. Nonetheless, other meta-analyses have indicated that there is minimal effect on muscle mass and a slight improvement in muscle strength in older adults [69,70]. The two previous meta-analyses also indicated disparities in what occurred to functional ability with ω-3 supplementation, with one indicating an improvement [69] and one indicating no effect [70]. Furthermore, narrative reviews on the topic have indicated that more evidence is needed to support the use of ω-3 supplementation strategies [71], the frequency and dosing pattern used to reduce the risk of sarcopenia needs to be delineated [72], and the anti-sarcopenic influence of ω-3 supplementation in older adults requires further clarification [73]. However, many older individuals are largely sedentary and have reduced amounts of physical activity, which makes it vital to find strategies, such as ω-3 intake, to improve muscle health in aging adults and reduce the healthcare impact this has on individuals and society [74].

## 5. Future Directions for Research

Upcoming research in this area should focus on the underlying mechanisms whereby ω-3 supplementation strategies may enhance skeletal muscle parameters in the absence of other interventions. This will provide clear information on the potential benefits (and possible drawbacks) of supplementing with ω-3 fatty acids. ω-3 fatty acid dosing is extremely variable between different study designs. Finding the optimal dose that could enhance skeletal muscle, particularly in aging individuals, is key to understanding the potential positive effects this nutrient could have. Also, more research in inflammatory non-communicable diseases (such as type-II diabetes, cardiovascular disease, autoimmune disease, and cancer) management by ω-3 supplementation strategies will likely reveal the underlying mechanisms whereby skeletal muscle could be enhanced in these conditions. 

## 6. Conclusions

The effects of ω-3 fatty acid intake alone on skeletal muscle form and function have not been fully elucidated. For now, it seems that the main mechanisms that ω-3s are involved with the aiding of skeletal muscle include (1) a decrease in inflammation, (2) enhancement of muscle protein synthesis, (3) alteration in the sensitivity of insulin, and (4) improvements in muscle satellite cell activity. Future work in this area will likely identify other underlying mechanisms at work in the positive effects that these fatty acids may have in enhancing and promoting skeletal muscle. 

## Data Availability

No new data were created with this review.
